# Supplementary material for: Genetic Etiology of Permanent Congenital Hypothyroidism in Korean Patients: A Whole-Exome Sequencing Study
Source: Int J Mol Sci. 2025 May 7;26(9):4465. doi: 10.3390/ijms26094465 (PMC12072708; doi:10.3390/ijms26094465)
Supplement: Supplementary file 1 [file ijms-26-04465-s001.zip › ijms-3554537-supplementary.pdf]

Supplementary Table 1. ACMG classification codes and population allele frequencies of variants identified in Korean patients with permanent congenital hypothyroidism

Patient No.	Gene	Variants (cDNA/protein)	ACMG classification	ACMG Codes	Total allele frequency	East Asian Allele frequency	KOVA allele frequency
1	*DUOX2*	c.714C>A (p.Tyr238Ter)	LP	PVS1, PM2	0.000005532	0	0.000322
2	*DUOX2*	c.1462G>A (p.Gly488Arg)	P	PS1, PM2, PM3, PP3, BP1	0.0001097	0.002429132	0.007013
3	*DUOX2*	c.3721A>T (p.Ile1241Phe)	VUS	PS1, PM2, PP3, BP1	6.195E-07	2.22787E-05	NA
4	*DUOX2*	c.1462G>A (p.Gly488Arg)	P	PS1, PM2, PM3, PP3, BP1	0.0001097	0.002429132	0.007013
	DUOX2	c.2654G>T (p.Arg885Leu)	LP	PS1, PM2, PM5, PP3, BP1	0.0001028	0.002852177	NA
5	*DUOX2*	c.2000del (p.Leu667ArgfsTer9)/	P	PVS1, PM2, PP5	0.00001859	0.000668568	NA
		c.3184+1G>A (p.?)	LP	PVS1, PM2	0.000001858	4.45633E-05	NA
6	*DUOX2*	c.1588A>T (p.Lys530Ter)	P	PVS1, PP5	0.0001357	0.004412551	0.000665
	DUOX2	c.4408C>T (p.Arg1470Trp)	VUS	PM2, PP3, BP1, BP6	0.00005886	0.00169416	0.001558
7	*DUOX2*	c.2654G>T (p.Arg885Leu)	LP	PS1, PM2, PM5, PP3, BP1	0.0001028	0.002852177	NA
	DUOX2	c.2428G>A (p.Glu810Lys)	VUS	PM2, PP3, BP1	0.00001797	0.000267404	0.000295
8	*DUOX2*	c.1462G>A (p.Gly488Arg)	P	PS1, PM2, PM3, PP3, BP1	0.0001097	0.002429132	0.007013
	TG	c.2359C>T (p.Arg787Ter)	P	PVS1, PM2, PP5	0.00003284	4.45494E-05	0.000294
9	*DUOX2*	c.3478_3480del (p.Leu1160del)	VUS	PM2, PM4, PP5	0.00002292	0.000802175	NA
	DUOX2	c.2048G>T (p.Arg683Leu)	VUS	PM2, PM5, PP3, BP1	0.00006939	0.002228363	0.000148
	TPO	c.2587G>A (p.Ala863Thr)	VUS	PS1, PM2, BP4	0.00004833	0.000289648	NA
10	*DUOX2*	c.3478_3480del (p.Leu1160del)	VUS	PM2, PM4, PP5	0.00002292	0.000802175	NA
	DUOXA2	c.738C>G (p.Tyr246Ter)	P	PVS1, PS3, PM2, PM3, PP5	0.00004115	0.001209406	NA
11	*DUOXA2*	c.413dup (p.Tyr138Ter)	P	PVS1, PM2, PP5	0.00005019	0.001715457	0.005061
12	*DUOXA2*	c.413dup (p.Tyr138Ter)	P	PVS1, PM2, PP5	0.00005019	0.001715457	0.005061
13	*DUOXA2*	c.738C>G (p.Tyr246Ter)	P	PVS1, PS3, PM2, PM3, PP5	0.00004115	0.001209406	NA
14	*TG*	c.2338C>T (p.Gln780Ter)	LP	PVS1, PM2	6.195E-07	2.22836E-05	0.000147

15	*TPO*	c.*63dup (p.?)	LP	PS3, PM2, PM3, PP5	0.00008449	0.002589748	NA
16	*TPO*	c.2017G>A (p.Glu673Lys)	VUS	PM2, PP3	0.00006697	0.000624053	0.002392
17	*TSHR*	c.1349G>A (p.Arg450His)	P	PS1, PS3, PM2, PM3, PM5, PP2, PP3	0.0001103	0.002652461	0.003030
18	*TSHR*	c.1411G>A (p.Ala471Thr)	VUS	PM2, PP2, PP3	0.00001425	6.683E-05	NA
19	*TSHR*	c.1349G>A (p.Arg450His)	P	PS1S, PS3, PM2, PM3, PM5, PP2, PP3	0.0001103	0.002652461	0.003030
	PAX8	c.898+1G>C (p.?)	VUS	PM2	6.361E-07	0	NA
20	*PAX8*	c.898+1G>C (p.?)	VUS	PM2	6.361E-07	0	NA

Abbreviations: P, pathogenic; LP, likely pathogenic; VUS, variant of uncertain significance; ACMG, American College of Medical Genetics and Genomics; KOVA, Korean Variant Archive; NA, not available.

ACMG classification codes are based on the 2015 ACMG-AMP guidelines.

Allele frequencies were derived from gnomAD v4.1, and East Asian- specific frequencies are included to approximate population-matched data in the absence of comprehensive Korean-specific datasets. KOVA allele frequencies were additionally reviewed where available.

Evidence strength codes include: **PVS,** Very strong evidence of pathogenicity; **PS,** Strong evidence; **PM,** Moderate evidence; **PP,** Supporting evidence; **BP/BS**, Supporting/strong evidence for benign classification.
